# Soulamarin Isolated from *Calophyllum brasiliense* (Clusiaceae) Induces Plasma Membrane Permeabilization of *Trypanosoma cruzi* and Mytochondrial Dysfunction

**DOI:** 10.1371/journal.pntd.0002556

**Published:** 2013-12-05

**Authors:** Alexandre Rea, Andre G. Tempone, Erika G. Pinto, Juliana T. Mesquita, Eliana Rodrigues, Luciana Grus M. Silva, Patricia Sartorelli, João Henrique G. Lago

**Affiliations:** 1 Instituto de Ciências Ambientais, Químicas e Farmacêuticas, Universidade Federal de São Paulo, Diadema, São Paulo, Brazil; 2 Departamento de Parasitologia, Instituto Adolfo Lutz, São Paulo, São Paulo, Brazil; 3 Instituto de Medicina Tropical, Universidade de São Paulo, São Paulo, São Paulo, Brazil; US Food and Drug Administration, United States of America

## Abstract

Chagas disease is caused by the parasitic protozoan *Trypanosoma cruzi*. It has high mortality as well as morbidity rates and usually affects the poorer sections of the population. The development of new, less harmful and more effective drugs is a promising research target, since current standard treatments are highly toxic and administered for long periods. Fractioning of methanol (MeOH) extract of the stem bark of *Calophyllum brasiliense* (Clusiaceae) resulted in the isolation of the coumarin soulamarin, which was characterized by one- and two-dimensional ^1^H- and ^13^C NMR spectroscopy as well as ESI mass spectrometry. All data obtained were consistent with a structure of 6-hydroxy-4-propyl-5-(3-hydroxy-2-methyl-1-oxobutyl)-6″,6″-dimethylpyrane-[2″,3″:8,7]-benzopyran-2-one for soulamarin. Colorimetric MTT assays showed that soulamarin induces trypanocidal effects, and is also active against trypomastigotes. Hemolytic activity tests showed that soulamarin is unable to induce any observable damage to erythrocytes (c_max._ = 1,300 µM). The lethal action of soulamarin against *T. cruzi* was investigated by using amino(4-(6-(amino(iminio)methyl)-1H-indol-2-yl)phenyl)methaniminium chloride (SYTOX Green and 1H,5H,11H,15H-Xantheno[2,3,4-ij:5,6,7-i′j′]diquinolizin-18-ium, 9-[4-(chloromethyl)phenyl]-2,3,6,7,12,13,16,17-octahydro-chloride (MitoTracker Red) as fluorimetric probes. With the former, soulamarin showed dose-dependent permeability of the plasma membrane, relative to fully permeable Triton X-100-treated parasites. Spectrofluorimetric and fluorescence microscopy with the latter revealed that soulamarin also induced a strong depolarization (ca. 97%) of the mitochondrial membrane potential. These data demonstrate that the lethal action of soulamarin towards *T. cruzi* involves damages to the plasma membrane of the parasite and mitochondrial dysfunction without the additional generation of reactive oxygen species, which may have also contributed to the death of the parasites. Considering the unique mitochondrion of *T. cruzi*, secondary metabolites of plants affecting the bioenergetic system as soulamarin may contribute as scaffolds for the design of novel and selective drug candidates for neglected diseases, mainly Chagas disease.

## Author Summary

Chagas disease is a parasitic protozoan that affects the poorest population in the world, causing a high mortality and morbidity. As a result of highly toxic and long-term treatments, the discovery of novel, safe and more efficacious drugs is essential. Natural products isolated from plants are commonly used as drug prototypes or precursors to treat parasitic diseases. As part of our investigation of bioactive compounds from Brazilian flora, the present study was undertaken in order to determine the antitrypanosomal effects of the soulamarin, a coumarin isolated from the stem bark of *Callophyllum brasiliense* (Clusiaceae), against *Trypanossoma cruzi*. This study moreover investigated the lethal action of soulamarin towards the parasite. Considering the obtained results, secondary metabolites of plants affecting the bioenergetic system as soulamarin may contribute as scaffolds for the design of novel and selective drug candidates for neglected diseases, mainly Chagas disease.

## Introduction

The tree *Calophyllum brasiliense* is known in Brazil as “Guanandi” or “Jacareúba”. It can reach up to 40 meters in high, 1–3 meters in diameter and is usually found in Brazil in the rain forest regions of the Amazon. Its stem bark is used in folk medicine to treat rheumatism, varicose veins, haemorrhoids and ulcers, whereas the leaves have anti-inflammatory properties [1]. Previous chemical studies on *C. brasiliense* resulted in the isolation of several interesting natural products, e.g. xantones, flavonoids, triterpenoids, and coumarins [2]. Some coumarins isolated from *C. brasiliense* displayed trypanocidal activity, but unfortunately, no information about the underlying mechanism was available [3].

The parasite *Trypanosoma cruzi* causes American trypanosomiasis or “Chagas disease”, which has high mortality and morbidity rates [4]. Chagas disease is common to the Americas, including Mexico and the South of the USA and has become a global public health problem [5]. Due to high levels of migration, the disease has already reached non-endemic countries. An estimated 10 million people are currently infected and 14,000 deaths per year are documented. In Brazil alone, over 6 million people are infected and approximately 6,000 deaths per year are registered. The migration of millions of Latin Americans to more developed countries such as e.g. the USA, accounts for approximately 300,000 chronically infected patients there [6]. More than a dozen infections acquired from blood transfusions or transplantations have been reported in several European countries, the USA, and Canada [7].

Nifurtimox (7–10 mg/kg/day) and benznidazole (5–7 mg/kg/day) are the two prevalent drugs, currently used in the treatment of Chagas disease. Unfortunately they suffer drawbacks from high levels of toxicity and long treatment periods (ca. 60 days) [8]. Nifurtimox, a nitrofuran, inhibits the ability of *T. cruzi* to deplete free radicals through the generation of a nitro-anion in the presence of oxygen. Benznidazole, a nitroimidazole, binds to the DNA, lipids and proteins of *T. cruzi* [9]. The average rate for successful cures among acute and recent cases is 80%, while it is less than 20% for chronic cases [10]. Several studies have identified numerous potential candidates for more effective and less toxic drugs. Amidines [11], [12], azoles [13], amiodarones [14], natural naphthoquinone derivatives and megazols [15] as well as calcium channel blockers [16] have been proposed, but clinically effective compounds still remain elusive. Therapeutic drug combinations have also been proposed as treatment strategies, e.g. benznidazole/nifurtimox, orbenznidazole/nifurtimoxin combination with antifungals which inhibit ergosterol in double or triple associations [17].

Natural products isolated from plants are commonly used as drug prototypes or precursors to treat parasitic diseases. Natural coumarins are an important class of plant products with antitrypanosomal activity [3], [18]. The coumarins mammea A/BA, A/BB, A/AA, A/BD and B/BA, isolated from *C. brasiliense* and *Mamea americana*, showed activity towards epimastigotes and trypomastigotes of *T. cruzi* for concentrations between 15 and 90 µg/mL [3]. Other coumarins isolated from the stem bark of *Kielmeyera albopunctata* showed *in vitro* activity against the trypomastigotes of *T. cruzi*, killing 80% of the parasites after 24 hours at 125 µg/mL [19].

Continuing the investigation of bioactive compounds from Brazilian flora, the present study was undertaken in order to determine the antitrypanosomal effects of soulamarin, which is the main compound isolated from the stem bark of *C. brasiliense*, against *T. cruzi*. This study moreover investigated the lethal action of soulamarin towards the parasite.

## Materials and Methods

### Chemical reagents and drugs

The compounds 3-[4,5-dimethylthiazol-2-yl]-2,5-diphenyltetrazolium bromide (MTT; Thiazol blue), mesoxalonitrile 4-trifluoromethoxyphenylhydrazone (FCCP), 4′,6-diamidino-2-phenylindole dihydrochloride (DAPI), sodium dodecyl sulfate (SDS), M-199 and RPMI-PR-1640 medium (without phenol red) as well as the NMR solvents CDCl_3_ and CD_3_OD were purchased from Sigma-Aldrich (USA). Dimethylsulfoxide (DMSO) was purchased from Merck (Brazil). 1H,5H,11H,15H-Xantheno[2,3,4-ij:5,6,7-i′j′]diquinolizin-18-ium, 9-[4-(chloromethyl)phenyl]-2,3,6,7,12,13,16,17-octahydro- chloride (MitoTracker Red CM-H_2_XROS), amino(4-(6-(amino(iminio)methyl)-1H-indol-2-yl)phenyl)methaniminium chloride (SYTOX Green) and 2′,7′-dichlorodihydrofluorescein diacetate (H_2_DCf-DA) were purchased from Molecular Probes (Invitrogen, Carlsbad, CA, USA). Silica gel (230–400 mesh) and Sephadex LH-20, used for column chromatography and analytical TLC (60 PF_254_), were either purchased from Merck (USA) or Sigma-Aldrich (USA). Benznidazole (2-nitroimidazole) was obtained from the *Laboratorio Farmaceutico do Estado de Pernambuco* – LAFEPE (Recife, Brazil).

### General experimental procedures

NMR spectra were recorded on a Bruker DRX-500 (^1^H: 500 MHz, ^13^C:125 MHz) spectrometer at ambient temperatures. Chemical shifts (δ) are reported in ppm and coupling constants (*J*) in Hz. All resonances were referenced to residual NMR solvent resonance. Low-resolution electrospray ionization mass spectra (LR-ESI-MS) were measured in positive mode on a Platform II-Micromass (quadrupole) mass spectrometer.

### Plant material

Samples of the stem bark of *C. brasiliense* were collected in the Amazonian rain forest of Brazil during September 2011. The authenticity of the plant material was verified by Dr. Eliana Rodrigues from ICAQF-UNIFESP. Sample specimens were deposited at the herbarium of the Instituto de Botânica - SEMA of São Paulo (SP, Brazil).

### Extraction and isolation of 6-Hydroxy-4-propyl-5-(3-hydroxy-2-methyl-1-oxobutyl)-6″,6″-dimethylpyrane-[2″,3″:8,7]-benzopyran-2-one (soulamarin)

**Figure 1:**
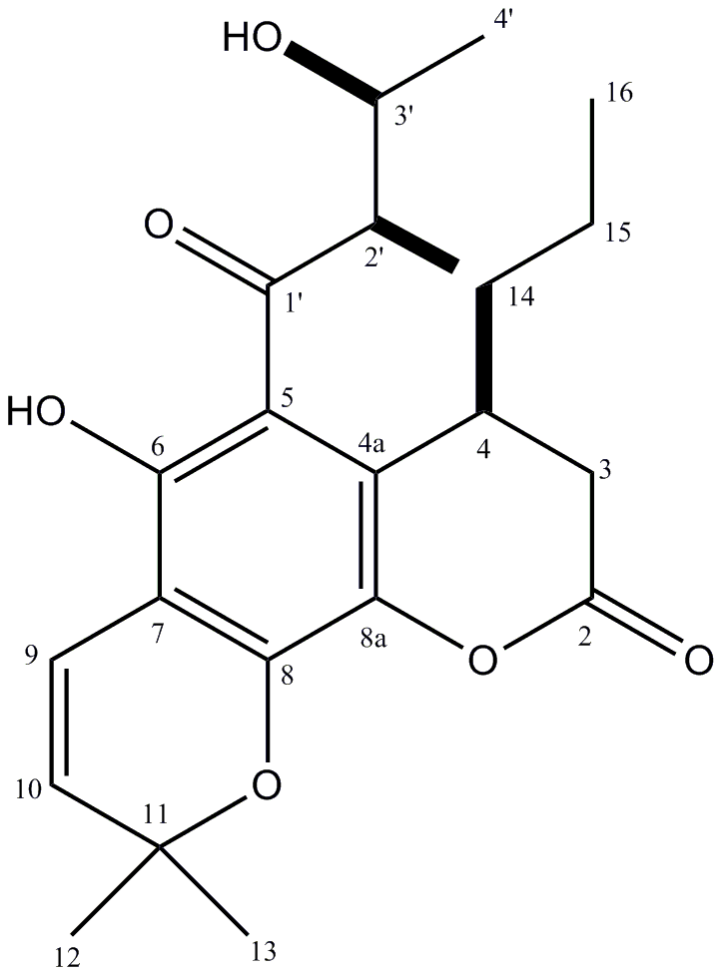
Chemical structure of soulamarin.

Dried and powdered stem bark samples of *C. brasiliense* (72 g) were washed exhaustively with hexane (10×500 mL) at room temperature in order to remove any residual fats. Subsequently, the plant material was extracted with MeOH (10×1 L) at room temperature. The combined organic fractions afforded, after removal of all solvents under reduced pressure, 4.7 g of crude residue. This crude extract was dissolved in MeOH:H_2_O (1∶2) and extracted with EtOAc. The removal of the solvent under reduced pressure resulted in the deposition of a residue (3.0 g), which was subsequently subjected to column chromatography (Sephadex LH-20) with MeOH as the eluent. Nine fractions (I–IX) were separated like this. Fraction III (1.31 g) was further purified by column chromatography over silica gel with a crude solvent gradient of hexane/EtOAc (starting with pure hexane and finishing with pure EtOAc). This second purification step afforded 544 mg of soulamarin (see Figure 1). ^1^H NMR (CDCl_3_/CD_3_OD) δ_H_ (ppm): 6.35 (d, *J* = 10.0 Hz, H-9), 5.26 (d, *J* = 10.0 Hz, H-10), 3.93 (m, H-3′), 3.48 (m, H-4), 2.4–2.5 (m, H-3a/H-3b), 2.30 (m, H-2′), 1.32 (m, H-15), 1.28 (d, *J* = 6.4 Hz, H-4′), 1.20 (s, H-12/H-13), 1.30 (m, H-14), 0.97 (d, *J* = 6.4 Hz, H-5′), 0.64 (t, *J* = 7.5 Hz, H-16). ^13^C NMR (CDCl_3_/CD_3_OD) δ_C_ (ppm): 199.1 (C-1′), 174.8 (C-2), 159.6 (C-8), 159.5 (C-8a), 156.4 (C-6), 125.5 (C-10), 115.2 (C-9), 109.4 (C-4a), 102.1 (C-7), 101.5 (C-5), 78.4 (C-3′), 77.4 (C-11), 45.3 (C-2′), 38.5 (C-3), 35.1 (C-15), 30.2 (C-4), 27.8 (C-12), 28.0 (C-13), 19.2 (C-4′), 20.4 (C-14), 13.7 (C-16), 10.0 (C-5′). LR-ESI-MS:*m/z* 389 [M+H]^+^ (calculated for C_22_H_28_O_6_: 388).

### Animals

Mice (swiss and BALB/c) were supplied by the animal breeding facility of the Adolfo Lutz institute (São Paulo, Brazil). Animals were kept in sterilized cages in a controlled environment, with water and food *ad libitum*. All experimental procedures were approved by the local ethics committee for animal use (CEUA-IAL/Pasteur 002/2011).

### Parasites and mammalian cells

In all *in vitro* assays, Y strains of *T. cruzi* trypomastigotes were used, which were kept at 37°C in LLC-MK2 (ATCC CCL 7) cells using RPMI-1640 medium with calf serum (2%) [20]. To keep the Y strains infective, trypomastigotes were also kept in swiss mice and regularly harvested from the bloodstream by heart puncture of infected animals at the peak of the parasitemia [21]. LLC-MK2 cells were maintained at 37°C in RPMI-1640 medium with fetal calf serum (10%) in an incubator (5% CO_2_ atmosphere).

### Antitrypanosomal activity

Trypomastigotes were counted in a hemocytometer (Neubauer) and deposited on a microplate (96 wells; 1×10^6^ cells/well). Subsequently, soulamarin was added to the cells in concentrations up to 386 µM and the cells were allowed to incubate for 24 hours at 37°C (5% CO_2_ atmosphere). Benznidazole was used as standard. Trypomastigote activity was based on the conversion of the soluble tetrazolium salt 3-[4,5-dimethylthiazol-2-yl]-2,5-diphenyltetrazolium bromide (MTT) into the insoluble formazan by mitochondrial enzymes. The extraction of formazan was carried out for 18 hours at 24°C with sodium dodecylsulfate (10%v/v; 100 µL/well) [22].

In order to determine the IC_50_ value for soulamarin against intracellular amastigotes, the method described by De Souza and co-workers [23] was used with minor modifications. Peritoneal macrophages were collected from the peritoneal cavity of BALB/c mice and deposited on a 16-well chamber slide (1×10^5^ cells/well) before being incubated for 24 hours at 37°C (5% CO_2_ atmosphere). Trypomastigotes from LLC-MK2-infected cultures were washed twice in RPMI-1640 medium, counted in a hemocytometer and added to the macrophages (parasite:macrophage ratio = 10∶1). After an incubation period of 18 hours at 37°C (5% CO_2_), residual free parasites were removed with two washings with medium. Soulamarin was subsequently incubated with infected macrophages (60 h, 37°C, 5% CO_2_) in a non-toxic concentration range between 3.01 and 386 µM. Benznidazole was used as a standard. At the end of the assay, slides were fixed with methanol and stained with Giemsa prior to counting under a light microscope. IC_50_ concentrations were obtained by counting 300 macrophages per well (in duplicate) and determining the number of amastigotes per infected macrophage.

### Cytotoxicity against mammalian cells

Peritoneal macrophages were collected from female BALB/C mice, seeded at 1×10^5^ cells/well in 96-well microplates and incubated with soulamarin for 72 h at 37°C in an incubator with 5% CO_2_. The viability of the cells was determined using MTT [16]. The data represent the mean of two independent assays (triplicates).

### Hemolytic activity

The hemolytic activity of soulamarin in concentrations up to 1,300 µM was evaluated from the erythrocytes of BALB/c mice [24]. A suspension (5%) of erythrocytesin PBS (phosphate buffered saline) was incubated with soulamarin at 25°C for 1 hour in a U-shaped microplate (96 wells). The absorption of the supernatant at 550 nm was recorded (FilterMax F5 Multi-Mode Microplate Reader-Molecular Devices).

### Spectrofluorimetric detection of the permeability of the cell membrane

Trypomastigotes were washed with PBS (phosphate buffered saline), deposited on a microplate (1×10^6^ cells/well) and incubated with SYTOX Green (1 µM) for 15 minutes at 24°C [25]. Soulamarin was added in three concentrations (IC_100_ = 386 µM, IC_50_ = 219 µM and IC_25_ = 103 µM) and the fluorescence was measured after 20, 40 and 60 minutes. The maximum permeability possible was observed with 0.1% Triton X-100 (positive control). The fluorescence intensity was determined using a fluorimetric microplate reader (FilterMax F5 Multi-Mode Microplate Reader-Molecular Devices) with excitation and emission wavelengths of 485 and 520 nm, respectively. Untreated trypomastigotes and 0.5% (v/v) DMSO-treated parasites were used as negative controls.

### Effect of soulamarin on the mitochondrial membrane potential

Trypomastigotes were washed with PBS, deposited on a microplate (2×10^6^ cells/well) and incubated with soulamarin (IC_50_ = 219 µM) for 60 minutes at 37°C. Then MitoTracker Red CM-H_2_XROS (500 nM) was added and the incubation was continued for 40 minutes in the dark. Cells were washed twice with BSS (Hank's buffered salt solution) and the fluorescence was measured using a fluorimetric microplate reader (FilterMax F5 Multi-Mode Microplate Reader-Molecular Devices) with excitation and emission wavelengths of 540 and 595 nm, respectively [26]. Untreated trypomastigotes and DMSO-treated parasites were used as negative controls. Mesoxalonitrile 4-trifluoromethoxyphenylhydrazone (FCCP; 10 µM) was used as a positive control [27]. For the fluorescence microscopy analysis, trypomastigotes were co-stained with 4′,6-diamidino-2-phenylindoledihydrochloride (DAPI; 10 µM) and examined at 1000× magnification. Merged images of blue (DAPI) and red (MitoTracker Red) images were obtained using the Nikon NIS - Elements AR software. A Nikon D-FL Epimicroscope equipped with a DS-U3 digital camera was used for the experiment.

### Analysis of reactive oxygen species (ROS)

Trypomastigotes (2×10^6^ cells/well) were washed in HBSS (Hanks Balanced Salt Solution) medium and incubated with soulamarin (IC_50_ = 219 µM) for 60 minutes at 37°C. To these cells 2′,7′-dichlorodihydrofluorescein diacetate (H_2_DCf-DA) was added (5 µM) and incubation was prolonged for 15 minutes. Then the fluorescence was measured using a fluorimetric microplate reader (FilterMax F5 Multi-Mode Microplate Reader-Molecular Devices) with excitation and emission wavelengths of 485 and 520 nm, respectively. Oligomycin (20 µM) was used as positive control [28]. Untreated trypomastigotes and parasites treated with DMSO were included as negative controls.

### Statistical analysis

Results are displayed as mean values ± standard deviations, which were obtained from at least two independent assays (n≥2). IC_50_ values were calculated from sigmoidal dose-response curves using the Graph Pad Prism 5.0 software. Confidence intervals of 95% are included in parentheses. The Student's *t*-test was used for significance testing (p<0.05) for all assays.

## Results

### Chemical characterization of soulamarin

The structure of soulamarin is shown in Figure 1. The assigned structure is consistent with the results obtained from NMR data and LR-ESI mass spectrum. The individual assignment of proton and carbon atoms was accomplished by 1D (^1^H, ^13^C) and 2D (HMQC, HMBC and NOESY) NMR measurements. The ^1^H NMR spectrum of soulamarin in CDCl_3_/CD_3_OD displayed two doublets at δ 6.35 and 5.26, with coupling constants of 10.0 Hz, which were assigned to H-9 and H-10. Together with the presence of a singlet at δ 1.20 (H-12 and H-13), these peaks suggested the presence of a chromene moiety [29]. The presence of a dihydrocoumarin segment was based on the multiplets at δ 2.4–2.5 (2H) and δ 3.48 (1H), which were assigned to H-3a/H-3b and H-4, respectively. The multiplets at δ 1.30 (H-14), 1.32 (H-15) and the triplet at δ0.64 (*J* = 7.5 Hz, H-16) were assigned to an *n*-propyl chain linked to C-4 [30]. The doublets at δ 0.97 and 1.28 (*J* = 6.4 Hz) were attributed to the methyl groups H-5′ and H-4′, while the multiplets at δ 2.30 and 3.93 were assigned to H-2′ and H-3′ of the isoprene moiety at C-5. The^13^C NMR spectra showed carbonyl carbons at δ 199.1 (C-1′) and 174.8 (C-2), as well as the sp^2^ carbon atoms of the chromene unit at δ 125.5 (C-10) and 115.2 (C-9). Resonances for aromatic carbon atoms were observed between δ 160 and 101, while the carbinol carbon atoms C-3′ and C-11 were observed at δ 78.4 and 77.4, respectively. Additional peaks, corresponding to an *n*-propyl group were observed at δ 35.1 (C-15), 20.4 (C-14) and 13.7 (C-16). Resonances corresponding to the isoprene unit were observed at δ 45.3 (C-2′), 19.2 (C-4′) and 10.0 (C-5′). The relative configurations of C-2′ and C-3′ were assigned by comparison of the NMR data with those reported for (2R*,3R*)- and (2R*,3S*)-3-hydroxy-2-methylpentanoic acid [31]. The configuration of C-4 was assigned as S*, due to the cross peaks between H-4_ax_ and H-3_eq_ as well as between H-4_ax_ and H-14, observed in the NOESY spectrum. All these results are consistent with a structure of 6-hydroxy-4-propyl-5-(3-hydroxy-2-methyl-1-oxobutyl)-6″,6″-dimethylpyrane-[2″,3″:8,7]-benzopyran-2-one (see Figure 1). The assigned structure was furthermore supported by comparison of our spectroscopic data with those reported in the literature [32].

### Antitrypanosomal, cytotoxicity and hemolytic activity of soulamarin

Soulamarin was incubated with trypomastigotes and the activity of cells was determined after 24 hours via MTT assay. Soulamarin thereby demonstrated activity against parasites, killing all the cells at the highest tested concentration. An IC_50_ value of 219.8 µM (95% confidence interval for 186.9–258.5 µM) was established (see Table 1). Benznidazole was used as standard against and resulted in an IC_50_ value of 440.7 µM (95% confidence interval for 272.4–478.4 µM). Soulamarin was also effective against intracellular amastigotes (IC_50_ = 210.1 µM; 95% confidence interval for 174.5–252.6 µM), while benznidazole showed an IC_50_ of 319.7 µM (95% confidence interval for 283.8–360.1 µM). The cytotoxicity of soulamarin was determined with peritoneal macrophages by the MTT assay. Soulamarin showed an IC_99_ value of 988.95 µM and IC_50_ value of 278.3 µM (95% confidence interval for 229.4–342.8 µM). The hemolytic activity was also examined, but soulamarin did not induce any observable hemolysis up to concentrations of 1,300 µM (Table 1).

**Table 1:** Evaluation of the 50% Inhibitory Concentration of soulamarin against trypomastigotes and intracellular amastigotes.

compound	IC_50_ (µM) trypomastigotes (95%CI)	IC_50_ (µM) amastigotes (95%CI)	Hemolytic (%) activity at 1,300 µM	IC_50_ (µM) Cytotoxicity (95%CI)
Soulamarin	219.8 (186.9–258.5)	210.1 (174.5–252.6)	0	278.3 (229.4–342.8)

The viability of trypomastigotes was determined with MTT at 550 nm and the hemolytic activity was determined at 550 nm. The efficacy of soulamarin against intracellular amastigotes was determined using light microscopy counting.

95%CI – 95% confidence interval; IC_50_ – 50% inhibitory concentration. IC_50_ of benznidazole against trypomastigotes - 440.7 µM (95%CI 272.4–478.4 µM) and against intracellular amastigotes - 319.7 µM (95%CI 283.8–360.1 µM).

### Modified permeability of the plasma membrane induced by soulamarin

**Figure 2:**
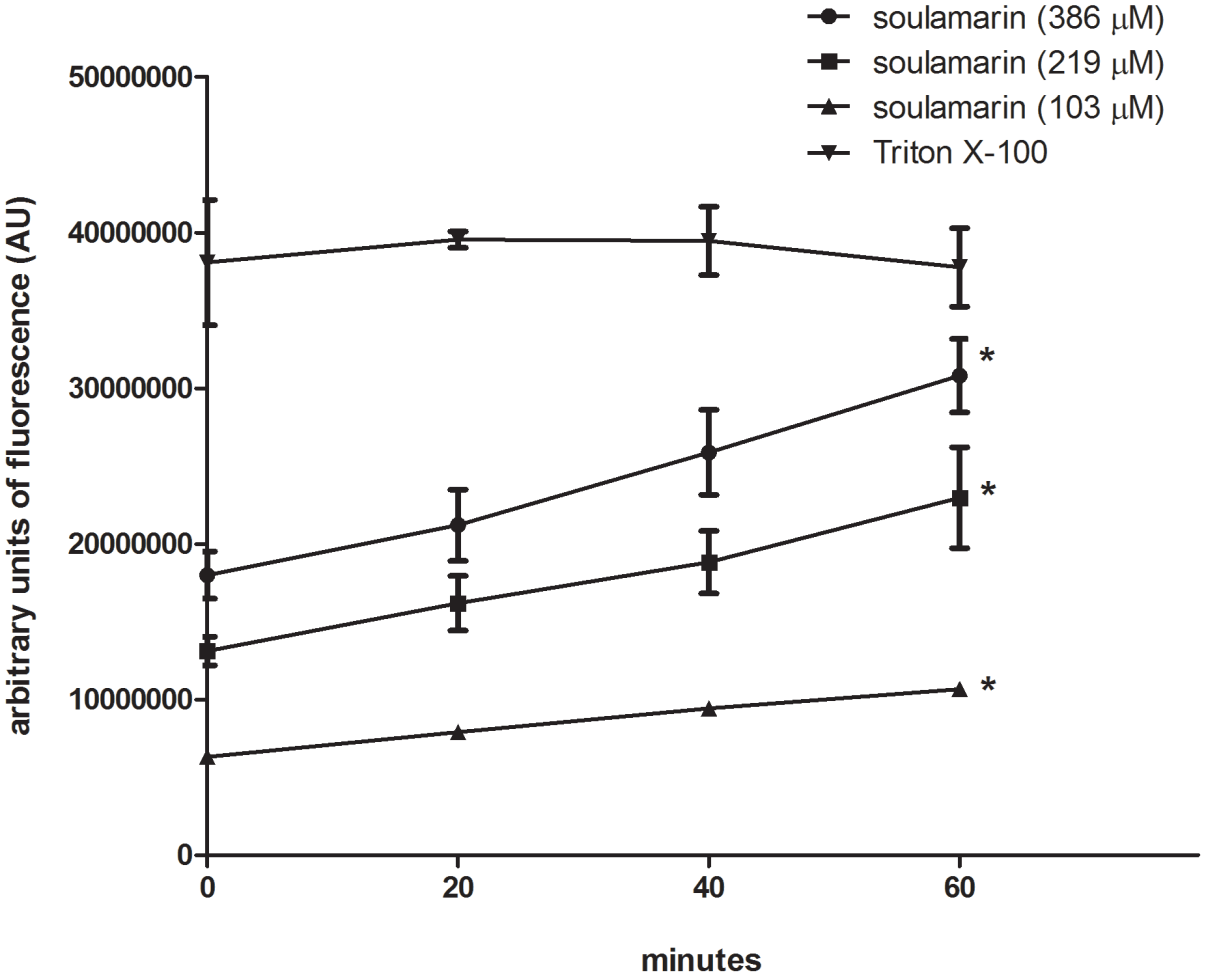
Fluorescence measurements (SYTOX Green) of *T. cruzi* after incubation with soulamarin, reflecting the modified permeability of the plasma membrane. Parasites were treated with soulamarin (IC_100_ = 386 µM, IC_50_ = 219 µM and IC_25_ = 103 µM) and compared to Triton X-100 (100% permeability = positive control) as well as an untreated negative control* (p<0.05).

Three different concentrations of soulamarin were incubated for up to 60 minutes with trypomastigotes and the permeability of the plasma membrane was examined by SYTOX Green assay. Soulamarin induced significant increased (p<0.05) fluorescence for all tested concentrations. Highest fluorescence intensities were observed after 60 minutes of incubation (Figure 2). Relative to fully permeabilized parasites (Triton X-100, 60 min), soulamarin induced the following percentages of permeability: i) 81% for IC_100_ = 386 µM (standard error of the mean SEM 6.2) (p<0.05); ii) 60% for IC_50_ = 219 µM (SEM 8.5); iii) 28% for IC_25_ = 103 µM (SEM 1.02). DMSO was used as internal control and resulted in lack of alteration.

### Soulamarin–induced depolarization of the mitochondrial membrane potential of *T. cruzi*

**Figure 3:**
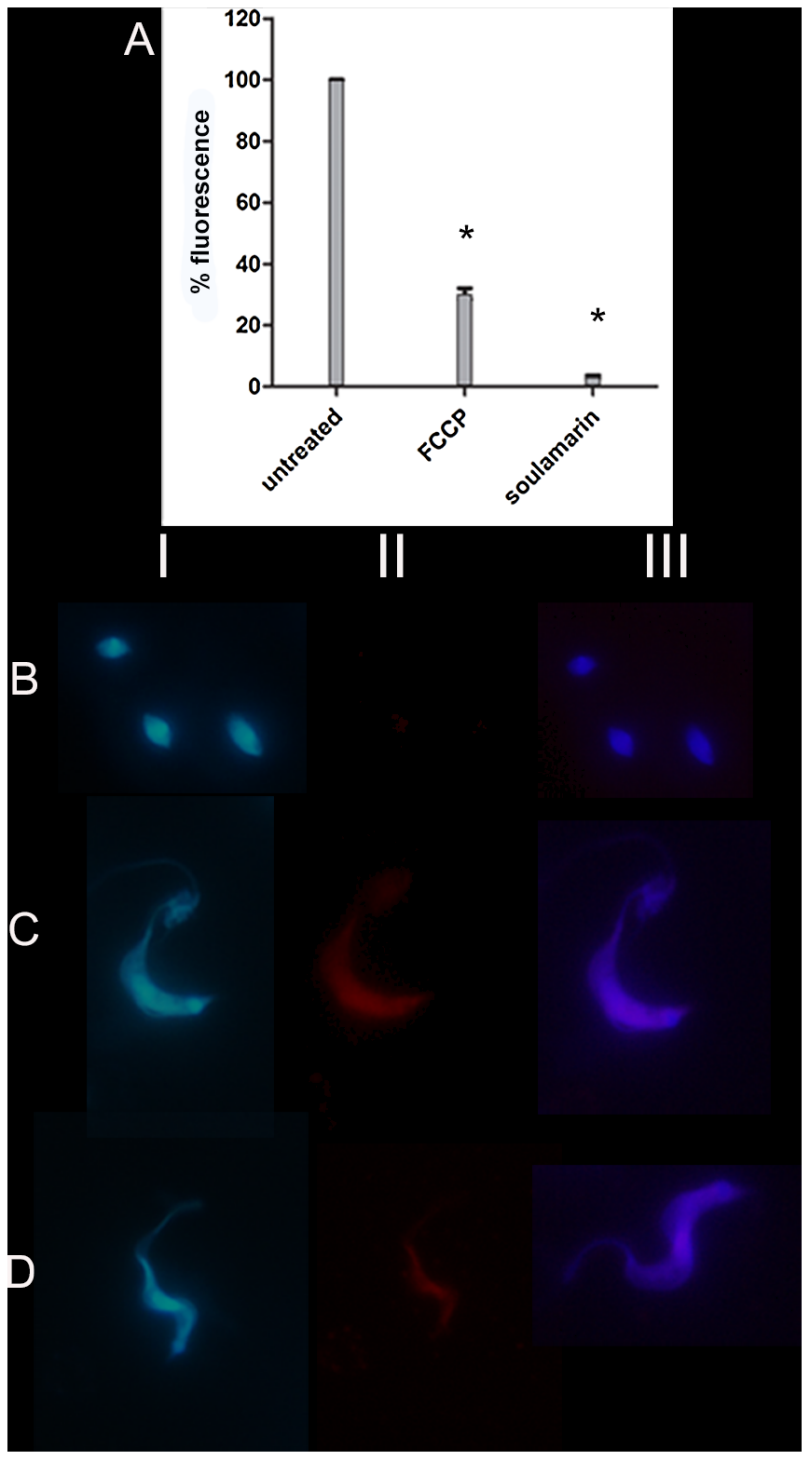
Mitochondrial membrane potential of *T. cruzi* trypomastigotes treated with soulamarin* (p<0.05). (**A**): Microplate spectrofluorimeter data showing soulamarin-treated *T. cruzi*, positive control (FCCP), and negative control (untreated cells). (**B–D**): Fluorescence microscope images, **B** - soulamarin-treated *T. cruzi*; **C** - untreated *T. cruzi* (negative control); **D** – FCCP (positive control). Panels **I** - images with blue fluorescence channel (DAPI); panels **II** - images with red fluorescence channel (MitoTracker Red); panels **III** - merged images (excitation and emission wavelengths of 540 and 595 nm; 1000×magnification).

Soulamarin was incubated with trypomastigotes (60 min) and the mitochondrial membrane potential was examined using MitoTracker Red. Spectrofluorimetric measurements indicated that soulamarin induced a significant (97%, p<0.05) decrease in fluorescence levels compared to untreated trypomastigotes (Figure 3A). The control group showed a typical mitochondrial membrane potential. FCCP was used as positive control, which reduced the fluorescence levels by 70% (p<0.05) relative to untreated parasites. Additional fluorescence microscopy experiments corroborated the spectrofluorimetric analysis, demonstrating a substantial reduction of fluorescence levels in soulamarin-treated parasites (Figure 3B), as wells as in FCCP (Figure 3D). Untreated parasites showed intense fluorescence levels of mitochondria after labeling with MitoTracker Red, which is consistent with a normal mitochondrial membrane potential (Figure 3C). Panels **I** represent images with blue fluorescence channel labeled with the fluorescent probe DAPI; panels **II** represent images with red fluorescence channel labeled with the fluorescent probe MitoTracker Red and panels **III**, represent the merged images.

### Analysis of reactive oxygen species (ROS)

Soulamarin was incubated with trypomastigotes and the up/down-regulation of ROS was examined using 2′,7′-dichlorodihydrofluorescein diacetate (H_2_DCf-DA). No changes in the production of ROS could be observed after 60 minutes. Oligomycin was used as positive control (100% ROS up-regulation). Untreated parasites were used as a negative control, showed a normal level of ROS production and were used for normalization (data not shown here).

## Discussion

Antitrypanosomal drugs targeting the bioenergetic metabolism as well as the plasma membrane have been considered as potentially chemotherapeutics for Chagas disease [33]. As part of an ongoing search aiming at the isolation of antiparasitic compounds from Brazilian plants [34]–[36], the coumarin 6-hydroxy-4-propyl-5-(3-hydroxy-2-methyl-1-oxobutyl)-6″,6″-dimethylpyrane-[2″,3″:8,7]-benzopyran-2-one (soulamarin) was isolated for the first time from the stem bark of *C. brasiliense* and characterized by ^1^H- and ^13^C-NMR analysis as well as by mass spectrometry. Ee and co-workers have recently isolated soulamarin from *C. soulattri*, but no biological activity was described [32]. To the best of our knowledge, this is the first time that an antiparasitic activity against *Trypanosoma cruzi* is reported for soulamarin. The comparable IC_50_ values of soulamarin and benznidazole, which is the currently drug in clinical use, suggests a similar effectiveness against trypomastigotes and intracellular amastigotes. In our assays, benznidazole showed an IC_50_ value of 440 µM against trypomastigotes. Different protocols have been described in literature for the evaluation of *T. cruzi* survival, resulting in different IC_50_ values. Such variations include: i) evaluation of cell lysis by light microscopy counting [37]; ii) spectrophotometric evaluation of MTT oxidation by mitochondrial dehydrogenases [38]; iii) culture-derived trypomatigotes and bloodstream trypomastigotes; iv) parasite strain; v) number parasites per well; vi) time of incubation with beznidazole, and vii) the source of the drug, which has been produced by different laboratories. Then, comparisons to other IC_50_ values of benznidazole should be carefully analyzed. Natural products and synthetic compounds affecting the biosynthesis [33] or the permeability of the plasma membrane [39] of *T. cruzi* have been identified as interesting targets for drug discovery studies. By way of using different fluorimetric probes (SYTOX Green, MitoTracker Red), we targeted in this study the mechanistic aspects on how soulamarin kills *T. cruzi* trypomastigotes. Our data demonstrated that soulamarin rapidly altered the permeability of the plasma membrane, resulting in a dose- and time-dependent influx of the vital dye SYTOX Green into the cell. Fluorescence levels were constant during the observation period (60 min) for all tested concentrations of soulamarin. At 386 µM (IC_100_), soulamarin induced the highest fluorescence intensity, which was close to the positive control Triton X-100, suggesting long-term effects on the membrane of the parasite. A similar effect was also observed at the lowest tested concentration (IC_25_ = 103 µM). Edelfosine, a synthetic lysophosphlipid drug has also been shown to induce alterations in the plasma membrane and mitochondria of *T. cruzi*, suggesting that these organelles could be potential targets [40]. However, it has also been proposed that a drug-induced change of the permeability of the plasma membrane is not necessarily accompanied by changes of the mitochondrial function. Digitonin for example, a natural glycoside obtained from *Digitalis* sp., has been shown to increase plasma membrane permeability in *T. cruzi*, without affecting the mitochondria [41].

The respiratory apparatus of protozoans typically displays a greater diversity in electron pathways compared to their host cells. The mitochondrion of the protozoan can be considered as a valuable drug target, because of its unique structure and function compared to mammalian cells [42]. The predominant physiological function of the mitochondrion is the generation of ATP by oxidative phosphorylation. Additional functions include the generation and detoxification of ROS, the involvement in some forms of apoptosis and the regulation of cytoplasmic and mitochondrial calcium [43]. Furthermore, a proper mitochondrial membrane potential is essential for the survival of cells and changes can result in a variety of consequence, such as the inhibition of the electron transport chain, the inhibition of ATP synthase, the stimulation of uncoupling proteins or the permeability of the inner membrane [44]. In our assays, soulamarin rapidly induced a depolarization of the mitochondrial membrane potential in trypomastigotes, resulting in a reduction of the fluorescence intensity by 97%, relative to the untreated group. This effect may have contributed to deleterious cellular damages associated with bioenergetic system. A similar, potent dose-dependent collapse of the mitochondrial membrane potential, resulting in the killing of *T. cruzi* parasites, has been reported for synthetic naphthofuranquinones [45].

Under physiological conditions, the oxidative phosphorylation involving an electron transport to pump hydrogen ions across the inner membrane, releases ROS, amounting to 3–5% of the total amount of oxygen consumed [46]. Under pathologic conditions, several pathways result in excessive ROS production, which causes - if not efficiently scavenged by the antioxidant system - oxidative stress. Proteins, lipids, and DNA are readily oxidized by ROS, resulting in dysfunction of vital physiological processes, oxidative damage, and cell death [47]. In our assays, despite the substantial depolarization of the mitochondrial membrane potential of *T. cruzi*, soulamarin induced no up-regulation of ROS compared to untreated trypomastigotes. Within the mitochondria, the primary site of ROS production is the electron transport chain, which involves four protein-associated complexes [48]. Several cellular enzyme systems are potential sources of ROS:NAD(P)H oxidase, xanthine oxidase, uncoupled endothelial nitric oxide synthase (eNOS), arachidonic acid metabolizing enzymes such as cytochrome P-450 enzymes, lipoxygenase and cyclooxygenase, as well as the mitochondrial respiratory chain. Considering that a large number of drugs, which affect mitochondria also contribute to an up-regulation of ROS [49]–[50], we propose that soulamarin could target *T. cruzi* mitochondria without affecting the enzymes mentioned above.

### Conclusion

Soulamarin was isolated for the first time from the stem bark of *C. brasiliense* and showed desirable anti-trypanosomal activity. Our results furthermore indicated that soulamarin-induced death in *T. cruzi* is associated with mitochondrial dysfunction and a modified permeability of the plasma membrane. Therefore, the natural product soulamarin could serve as a scaffold for the development of selective new drugs against neglected diseases, in particular Chagas disease.

## References

[1] Cechinel-Filho V, Meyre-Silva C, Niero R (2009) Chemical and pharmacological aspects of the genus *Calophyllum* . Chemistry & Biodiversity 6: 313–327. 19319867 10.1002/cbdv.200800082

[2] Noldin VF, Isaias DB, Cechinel-Filho V (2006) Gênero *Calophyllum*: importância química e farmacológica. Química Nova 29: 549–554.

[3] Reyes-Chilpa R, Estrada-Muñiz E, Vega-Avila E, Abe F, Kinjo J, et al (2008) Trypanocidal constituents in plants: 7. Mammea-type coumarins, Memorias do Instituto Oswaldo Cruz 103: 431–436. 18797754 10.1590/s0074-02762008000500004

[4] Rassi A Jr, Rassi A, Rezende JM (2012) American trypanosomiasis (Chagas disease). Infectious Disease Clinics of North America 26: 275–291. 22632639 10.1016/j.idc.2012.03.002

[5] WHO Chagas disease (American trypanosomiasis) fact sheet (revised in June 2010). Weekly Epidemiological Record 85: 334–336. 20726172

[6] Bern C, Montgomery S (2009) An estimate of the burden of Chagas disease in the United States. Clinical Infectious Diseases 49: e52–54. 19640226 10.1086/605091

[7] Tempone AG, Sartorelli P, Mady C, Fernandes F (2007) Natural products to anti-trypanosomal drugs: an overview of new drug prototypes for American trypanosomiasis. Cardiovascular & Hematological Agents in Medicinal Chemistry 5: 222–235. 17630949 10.2174/187152507781058726

[8] Bernardes LSC, Zani CL, Carvalho I (2013) Trypanosomatidae diseases: from the current therapy to the efficacious role of Trypanothione reductase in drug Discovery. Current Medicinal Chemistry 20: 2673–2696. 23410156 10.2174/0929867311320210005

[9] Dias-de-Toranzo EG, Castro JA, Franke-de-Cazzulo BM, Cazzulo JJ (1988) Interaction of Benznidazole reative metabolism with nuclear and kinoplastic DNA, protein and lipids from *Trypanosoma cruzi* . Experientia 44: 880–881. 3053234 10.1007/BF01941187

[10] Coura JR, Borges-Pereira J (2011) Chronic phase of Chagas disease: why should it be treated? A comprehensive review. Memorias do Instituto Oswaldo Cruz 106: 641–645. 22012216 10.1590/s0074-02762011000600001

[11] Soeiro MN, Werbovetz K, Boykin DW, Wilson WD, Wang MZ, et al (2013) Novel amidines and analogues as promising agents against intracellular parasites: a systematic review. Parasitology 140: 929–951. 23561006 10.1017/S0031182013000292 PMC3815587

[12] Daliry A, Pires MQ, Silva CF, Pacheco RS, Munde M, et al (2011) The trypanocidal activity of amidine compounds does not correlate with their binding affinity to *Trypanosoma cruzi* kinetoplast DNA. Antimicrobial Agents and Chemotherapy 55: 4765–4773. 21807972 10.1128/AAC.00229-11 PMC3186963

[13] Buckner FS, Urbina JA (2012) Recent developments in sterol 14-demethylase inhibitors for Chagas disease. International Journal for Parasitology: Drugs and Drug Resistance 2: 236–242. 23277882 10.1016/j.ijpddr.2011.12.002 PMC3531554

[14] Veiga-Santos P, Barrias ES, Santos JF, de Barros Moreira TL, de Carvalho TM, et al (2012) Effects of amiodarone and posaconazole on the growth and ultrastructure of *Trypanosoma cruzi* . International Journal of Antimicrobial Agents 40: 61–71. 22591838 10.1016/j.ijantimicag.2012.03.009

[15] Soeiro M de N, de Castro SL (2011) Screening of potential anti-*Trypanosoma cruzi* candidates: *in vitro* and *in vivo* studies. The Open Medicinal Chemistry Journal 5: 21–30. 21629508 10.2174/1874104501105010021 PMC3103897

[16] Reimão JQ, Scotti MT, Tempone AG (2010) Anti-leishmanial and anti-trypanosomal activities of 1,4-dihydropyridines: *in vitro* evaluation and structure-activity relationship study. Bioorganic & Medicinal Chemistry 18: 8044–8053. 20934347 10.1016/j.bmc.2010.09.015

[17] Coura JR, Borges-Pereira J (2012) Chagas disease. What is known and what should be improved: a systemic review. Revista da Sociedade Brasileira de Medicina Tropical 45: 286–296. 22760123 10.1590/s0037-86822012000300002

[18] Pérez-Cruz F, Serra S, Delogu G, Lapier M, Maya JD, et al (2012) Antitrypanosomal and antioxidant properties of 4-hydroxycoumarins derivatives. Bioorganic & Medicinal Chemistry Letters 22: 5569–5573. 22832320 10.1016/j.bmcl.2012.07.013

[19] Scio E, Ribeiro A, Alves TM, Romanha AJ, Shin YG, et al (2003) New bioactive coumarins from *Kielmeyera albopunctata* . Journal of Natural Products 66: 634–637. 12762797 10.1021/np020597r

[20] Bettiol E, Samanovic M, Murkin AS, Raper J, Buckner F, et al (2009) Identification of three classes of heteroaromatic compounds with activity against intracellular *Trypanosoma cruzi* by chemical library screening. PLoSNeglected Tropical Diseases 3: e384. 10.1371/journal.pntd.0000384 PMC2639639 19238193

[21] Meirelles MN, Chiari E, de Souza W (1982) Interaction of bloodstream, tissue culture-derived and axenic culture-derived trypomastigotes of *Trypanosoma cruzi* with macrophages. Acta Tropica 39: 195–203. 6128888

[22] Lane JE, Ribeiro-Rodrigues R, Suarez CC, Bogitsh BJ, Jones MM, et al (1996) *In vitro* trypanocidal activity of tetraethylthiuram disulfide and sodium diethylamine-N-carbodithioate on *Trypanosoma cruzi* . American Society of Tropical Medicine and Hygiene 55: 263–266. 10.4269/ajtmh.1996.55.263 8842112

[23] De Souza EM, da Silva PB, Nefertiti AS, Ismail MA, Arafa RK, et al (2011) Trypanocidal activity and selectivity *in vitro* of aromatic amidine compounds upon bloodstream and intracellular forms of *Trypanosoma cruzi* . Experimental Parasitology 127: 429–435. 20971106 10.1016/j.exppara.2010.10.010

[24] Moreira DR, Lima-Leite AC, Pinheiro-Ferreira PM, da Costa PM, Costa-Lotufo LV, et al (2007) Synthesis and antitumour evaluation of peptidyl-like derivatives containing the 1,3-benzodioxole system. European Journal of Medicinal Chemistry 42: 351–357. 17175071 10.1016/j.ejmech.2006.10.007

[25] Mangoni ML, Saugar JM, Dellisanti M, Barra D, Simmaco M, et al (2005) Temporins, small antimicrobial peptides with leishmanicidal activity. The Journal of Biological Chemistry 280: 984–990. 15513914 10.1074/jbc.M410795200

[26] Williams RA, Smith TK, Cull B, Mottram JC, Coombs GH (2012) ATG5 is essential for ATG8-dependent autophagy and mitochondrial homeostasis in *Leishmania major* . PLoS Pathogens 8: e1002695. 22615560 10.1371/journal.ppat.1002695 PMC3355087

[27] Chen L, Liu T, Tran A, Lu X, Tomilov AA, et al (2010) OPA1 mutation and late-onset cardiomyopathy: mitochondrial dysfunction and mtDNA instability. Journal of American Heart Association 1: e003012. 10.1161/JAHA.112.003012 PMC3541627 23316298

[28] Ribeiro GA, Cunha-Júnior EF, Pinheiro RO, da-Silva SA, Canto-Cavalheiro MM, et al (2013) LQB-118, na orally active pterocarpanquinone, induces selective oxidative stress and apoptosis in *Leishmania amazonensis* . Journal of Antimicrobial Chemotherapy 68: 789–799. 23288404 10.1093/jac/dks498

[29] Kitamura ROS, Romoff P, Young MCM, Kato MJ, Lago JHG (2006) Chromenes from *Peperomia serpens* (Sw.) Loudon (Piperaceae). Phytochemistry 67: 2398–2402. 16973191 10.1016/j.phytochem.2006.08.007

[30] Brenzan MA, Santos AO, Nakamura CV, Dias-Filho BP, Ueda-Nakamura T, et al (2012) Effects of (-) mammea A/BB isolated from *Calophyllum brasiliense* laves and derivatives on mitocondrial membrane of *Leishmania amazonensis* . Phytomedicine 19: 223–230. 22285848 10.1016/j.phymed.2011.10.008

[31] Brown JM, Evans PL, James AP (1993) Direct homogeneous hydrogenation: methyl anti-3-hydroxy-2-methylpentanoate. Organic Syntheses Collective 8: 420–425.

[32] Ee GCL, Mah SH, Teh SS, Rahmani M, Go R, et al (2011) Soulamarin, a new coumarin from stem bark of *Calophyllum soulattri* . Molecules 16: 9721–9727. 22113580 10.3390/molecules16119721 PMC6264249

[33] Kessler RL, Soares MJ, Probst CM, Krieger MA (2013) *Trypanosoma cruzi* response to sterol biosynthesis inhibitors: morphophysiological alterations leading to cell death. PLoS One 8: e55497. 23383204 10.1371/journal.pone.0055497 PMC3561218

[34] Grecco SS, Reimão JQ, Tempone AG, Sartorelli P, Romoff P, et al (2010) Isolation of an antileishmanial and antitrypanosomal flavanone from the leaves of *Baccharis retusa* DC. (Asteraceae). Parasitology Research 106: 1245–1248. 20165875 10.1007/s00436-010-1771-8

[35] Grecco SS, Reimão JQ, Tempone AG, Sartorelli P, Cunha RLOR, et al (2012) *In vitro* antileishmanial and antitrypanosomal activities of flavanones from *Baccharis retusa* DC. (Asteraceae). Experimental Parasitology 130: 141–145. 22143090 10.1016/j.exppara.2011.11.002

[36] Morais TR, Romoff P, Favero OA, Reimão JQ, Lourenço WC, et al (2012) Anti-malarial, anti-trypanosomal and anti-leishmanial activities of jacaranone isolated from *Pentacalia desiderabilis* (Vell.) Cuatrec. (Asteraceae). Parasitology Research 110: 95–101. 21614544 10.1007/s00436-011-2454-9

[37] Ferreira SB, Salomão K, de Carvalho da Silva F, Pinto AV, Kaiser CR, et al (2011) Synthesis and anti-*Trypanosoma cruzi* activity of β-lapachone analogues. European Journal of Medicinal Chemistry 46: 3071–3077. 21450374 10.1016/j.ejmech.2011.03.012

[38] Gehrke SS, Pinto EG, Steverding D, Pleban K, Tempone AG, et al (2013) Conjugation to 4-aminoquinoline improves the anti-trypanosomal activity of Deferiprone-type iron chelators. Bioorganic Medicinal Chemistry 21: 805–813. 23266185 10.1016/j.bmc.2012.11.009

[39] Fernandes MP, Inada NM, Chiaratti MR, Araújo FF, Meirelles FV, et al (2010) Mechanism of *Trypanosoma cruzi* death induced by *Cratyliamollis* seed lectin. Journal of Bioenergetic Biomembranes 42: 69–78. 10.1007/s10863-010-9268-9 20155390

[40] Santa-Rita RM, Barbosa HS, de Castro SL (2006) Ultra-structural analysis of edelfosine-treated trypomastigotes and amastigotes of *Trypanosoma cruzi* . Parasitology Research 100: 187–190. 16855821 10.1007/s00436-006-0250-8

[41] Vercesi AE, Bernardes CF, Hoffmann ME, Gadelha FR, Docampo R (1991) Digitonin permeabilization does not affect mitochondrial function and allows the determination of the mitochondrial membrane potential of *Trypanosoma cruzi in situ* . Journal of Biological Chemistry 266: 14431–14434. 1860850

[42] Sen N, Majumder HK (2008) Mitochondrion of protozoan parasite emerges as potent therapeutic target: exciting drugs are on the horizon,. Current Pharmaceutical Design 14: 839–846. 18473833 10.2174/138161208784041024

[43] Brand MD, Nicholls DG (2011) Assessing mitochondrial dysfunction in cells. Biochemical Journal 435: 297–312. 21726199 10.1042/BJ20110162 PMC3076726

[44] Fidalgo LM, Gille L (2011) Mitochondria and trypanosomatids: targets and drugs. Pharmaceutical Research 28: 2758–2770. 21935742 10.1007/s11095-011-0586-3

[45] Menna-Barreto RF, Goncalves RL, Costa EM, Silva RS, Pinto AV, et al (2009) The effects on *Trypanosoma cruzi* of novel synthetic naphthoquinones are mediated by mitochondrial dysfunction. Free Radical Biology & Medicine 47: 644–653. 19501647 10.1016/j.freeradbiomed.2009.06.004

[46] Boonstra J, Post JA (2004) Molecular events associated with reactive oxygen species and cell cycle progression in mammalian cells. Gene 4: 1–13. 10.1016/j.gene.2004.04.032 15276197

[47] Gupta S, Bhatia V, Wen JJ, Wu Y, Huang MH, et al (2009) *Trypanosoma cruzi* infection disturbs mitochondrial membrane potential and ROS production rate in cardiomyocytes. Free Radical Biolology & Medicine 47: 1414–1421. 10.1016/j.freeradbiomed.2009.08.008 PMC2767388 19686837

[48] Zhang DX, Gutterman DD (2007) Mitochondrial reactive oxygen species-mediated signaling in endothelial cells. Heart and Circulatory Physiology: American Journal of Physiology 292: H2023–2031. 17237240 10.1152/ajpheart.01283.2006

[49] Carvalho L, Luque-Ortega JR, Manzano JI, Castanys S, Rivas L, et al (2010) Tafenoquine, anantiplasmodial 8-aminoquinoline, targets *Leishmania* respiratory complex III and induces apoptosis. Antimicrobial Agents Chemotherapy 54: 5344–5351. 20837758 10.1128/AAC.00790-10 PMC2981264

[50] Roy A, Ganguly A, Bose-Dasgupta S, Das BB, Pal C, et al (2008) Mitochondria-dependent reactive oxygen species-mediated programmed cell death induced by 3,3′-diindolylmethane through inhibition of F0F1-ATP synthase in unicellular protozoan parasite *Leishmania donovani* . Molecular Pharmacology 74: 1292–1307. 18703668 10.1124/mol.108.050161

